# Supplementary material for: U0126 Compound Triggers Thermogenic Differentiation in Preadipocytes via ERK-AMPK Signaling Axis
Source: Int J Mol Sci. 2023 Apr 28;24(9):7987. doi: 10.3390/ijms24097987 (PMC10178890; doi:10.3390/ijms24097987)
Supplement: Supplementary file 1 [file ijms-24-07987-s001.zip › ijms-2298331-supplementary.pdf]

Supplementary Figure S1

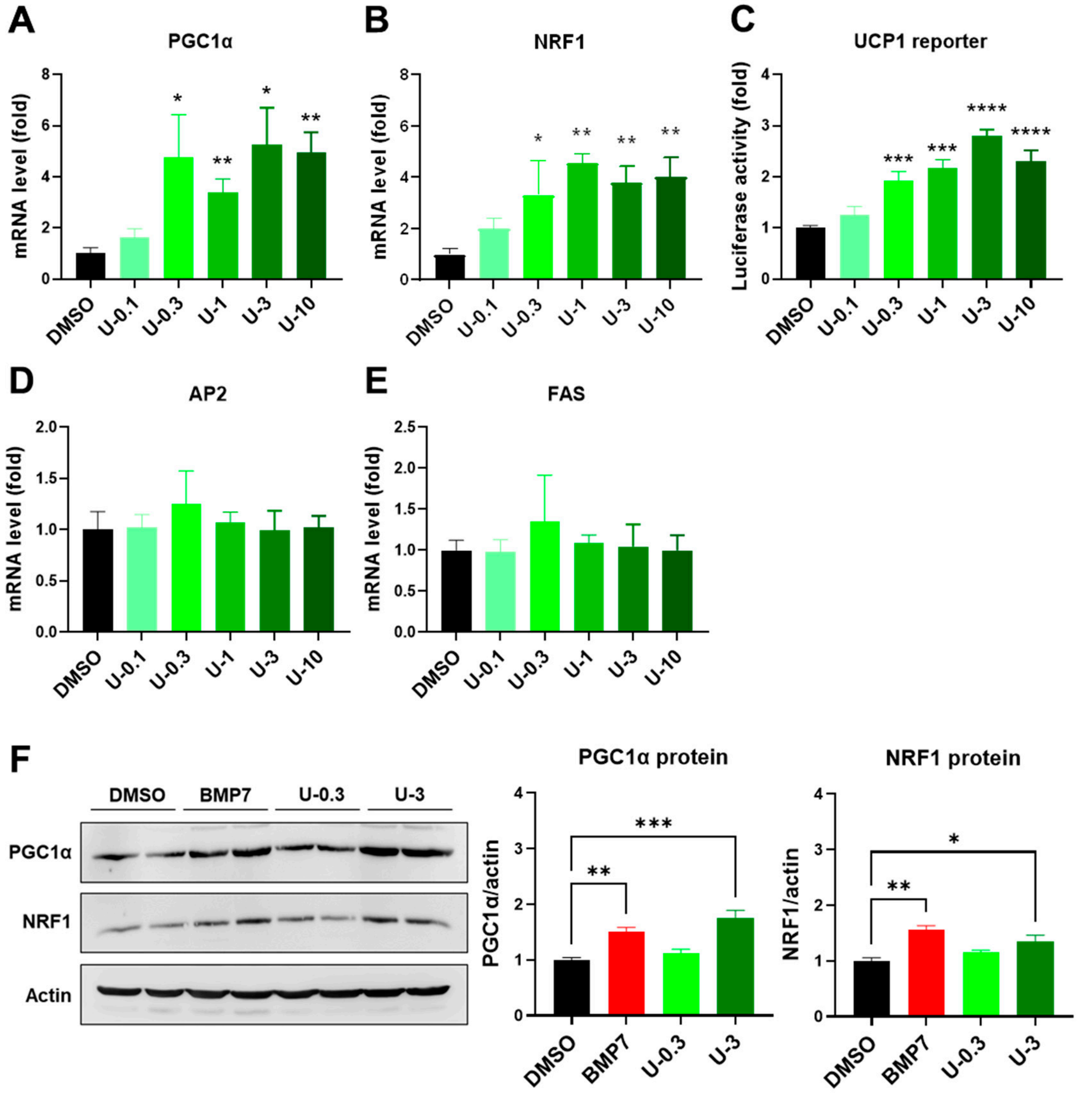

Supplementary Figure S1. U0126 pretreatment upregulates thermogenic genes after adipogenesis of human white preadipocytes. Human white preadipocytes were pretreated with different concentrations (0, 0.1, 0.3, 1, 3 and 10 μM) of U0126 (DMSO, U-0.1, U-0.3, U-1, U-3 and U-10) or BMP7 (3.3 nM) for 6 days. Preadipocytes underwent adipogenic differentiation for 12 days. (**A**-**E**) The mRNA levels of different genes and UCP1 reporter assay were measured. (**F**) The protein levels of PGC1α and NRF1 were measured by Western blot. The qualification of band intensity was analyzed, Unpaired Student’s t tests compared to DMSO group.

Supplementary Figure S2

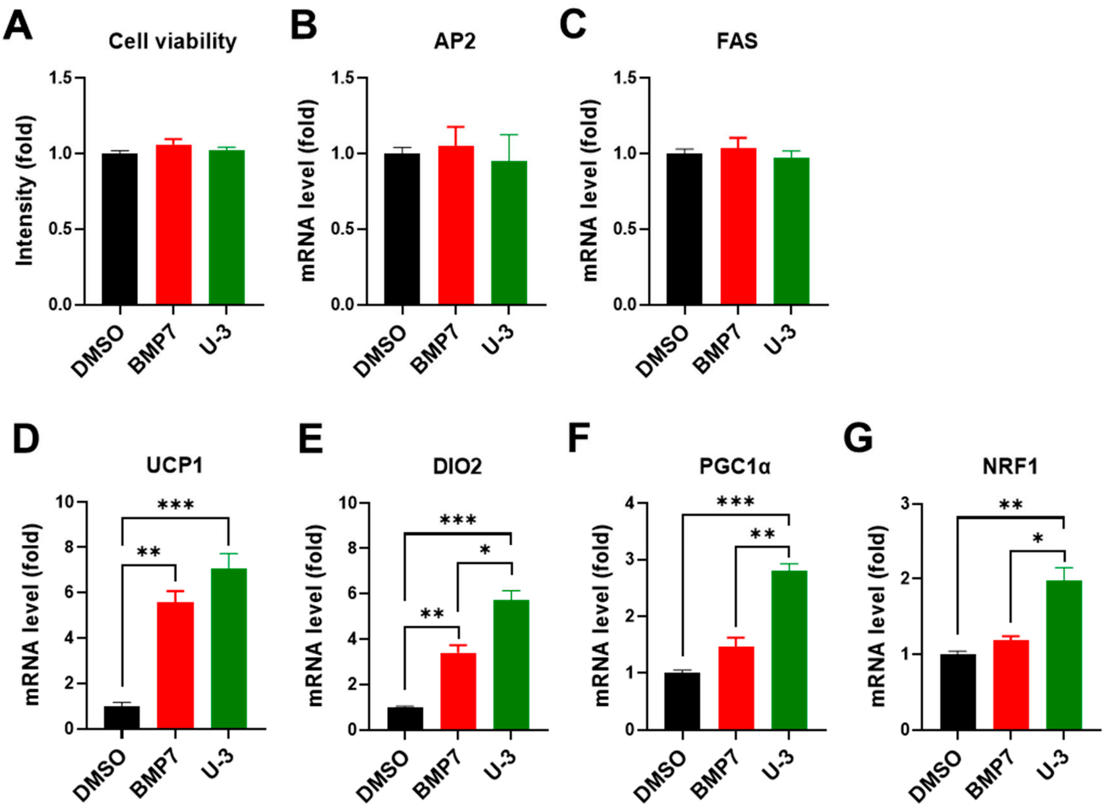

Supplementary Figure S2. U0126 pretreatment upregulates thermogenic genes after adipogenesis of human brown preadipocytes. (**A**) Human brown preadipocytes were pretreated with 3.3 nM of BMP7 or 3 μM of U0126 (U-3) for 6 days. The cell viability was measured. (**B**-**G**) After BMP7 or U0126 pretreatment, preadipocytes underwent adipogenic differentiation for 12 days. The mRNA levels of different genes were measured. Unpaired Student’s t tests compared to DMSO group.

Supplementary Table S1. Candidate compounds identified by CMAP.

Rank	Compound	Score	Target
1	U0126	85.9	Mitogen-activated protein kinase kinase (MEK)
2	Tadalafil	84.6	Phosphodiesterase (PDE)
3	Ingenol	82.4	Protein kinase C (PKD)
4	Fexaramine	80.1	Farnesoid X receptor (FXR)
5	Fenretinide	77.3	Retinoic acid receptor (RAR)
6	Trametinib	76.9	Mitogen-activated protein kinase kinase (MEK)
7	Naproxen	74.3	Cyclooxygenase (COX)
8	ZM-447439	72.8	Aurora kinase
9	Butein	71.9	Epidermal growth factor receptor (EGFR)
10	Tamibarotene	70.4	Retinoic acid receptor (RAR)

Supplementary Table S2. Primer sequences.

	Gene	Forward primer	Reverse primer
Human	18S rRNA	TCAACTTTCGATGGTAGTCGCCGT	TCCTTGGATGTGGTAGCCGTTTCT
	UCP1	ACCGCAGGGAAAGAAACAGC	TCAGATTGGGAGTAGTCCCT
	AP2	ACTGGGCCAGGAATTTGACGAAGT	TCTCGTGGAAGTGACGCCTTTCAT
	FAS	GCATCTGGACCCTCCTACCT	TCCTCAATTCCAATCCCTTG
	DIO2	TGTGCAGGCAGACAGACC	AAAGTCAAGAAGGTGGCATGTGGC
	PGC1α	AGTGGTGCAGTGACCAATCA	CTGCTAGCAAGTTTGCCTCA
	NRF1	AGGAACACGGAGTGACCCAA	TATGCTCGGTGTAAGTAGCCA

Supplementary Table S3. Antibodies.

Antibody	Vendor	Catalog no.
UCP1	abcam	ab155117
PGC1α	abcam	ab188102
NRF1	abcam	ab55744
AMPKα	Cell Signaling Technology	5831
Phosphorylated AMPKα (Thr172)	Cell Signaling Technology	2535
p44/42 MAPK (ERK1/2)	Cell Signaling Technology	4695
Phospho-p44/42 MAPK (ERK1/2) (Thr202/Tyr204)	Cell Signaling Technology	4370
Actin	Merck Millipore	MAB1501
β tubulin	Cell Signaling Technology	2146
